# Relationship between severe obesity and gut inflammation in children: what's next?

**DOI:** 10.1186/1824-7288-36-66

**Published:** 2010-10-01

**Authors:** Maria Immacolata Spagnuolo, Maria Pia Cicalese, Maria Angela Caiazzo, Adriana Franzese, Veronica Squeglia, Luca Rosario Assante, Giuliana Valerio, Rossella Merone, Alfredo Guarino

**Affiliations:** 1 Department of Paediatrics University Federico II, Naples, Italy; 2 School of Movement Sciences (DiSiST), Parthenope University, Naples, Italy

## Abstract

**Background:**

Preliminary evidence suggests an association between obesity and gut inflammation.

**Aims:**

To evaluate the frequency of glucose abnormalities and their correlation with systemic and intestinal inflammation in severely obese children.

**Patients and Methods:**

Thirty-four children (25 males; median age 10.8 ± 3.4 yrs) with severe obesity (BMI >95%) were screened for diabetes with oral glucose tolerance test (OGTT), systemic inflammation with C-reactive protein (CRP) and gut inflammation with rectal nitric oxide (NO) and faecal calprotectin.

**Results:**

BMI ranged from 23 to 44 kg/m^2^, and BMI z-score between 2.08 e 4.93 (median 2.69 ± 0.53). Glucose abnormalities were documented in 71% of patients: type 2 diabetes in 29%, impaired fasting glucose (IFG) in 58%, and impaired glucose tolerance (IGT) in 37.5%. Thirty-one patients (91%) were hyperinsulinemic. CRP was increased in 73.5% with a correlation between BMI z-score and CRP (p 0.03). Faecal calprotectin was increased in 47% patients (mean 77 ± 68), and in 50% of children with abnormal glucose metabolism (mean 76 ± 68 μg/g), with a correlation with increasing BMI z-score. NO was pathological in 88%, and in 87.5% of glucose impairment (mean 6.8 ± 5 μM).

**Conclusions:**

In this study, the prevalence of glucose abnormalities in obese children is higher than in other series; furthermore, a correlation is present between markers of systemic and intestinal inflammation and glucose abnormalities.

## Introduction

The global escalation of childhood obesity is a major concern, as excessive adiposity is believed to be the root cause of leading metabolic and cardiovascular diseases and related mortality. Widely prevalent in obese adults, obesity-related co-morbidities are beginning to surface in obese children as well [1,2], and we can expect a dramatic increase in adolescents and young adults afflicted with glucose intolerance, hypertension, dyslipidemia, non-alcoholic fatty liver disease (NAFLD) and ischemic heart disease and cancer in the very near future [3,4].

The prevalence of the metabolic syndrome increases with the severity of obesity and reaches 50% in severely obese youngsters [2]. Each half-unit increase in the body mass index, converted to a z score, is associated with an increase in the risk of the metabolic syndrome among overweight and obese subjects [5]. Mounting evidence suggests that insulin resistance resulting from obesity is pivotal to the pathogenesis of these metabolic consequences [6,7].

Obesity is associated with a chronic low-grade inflammation [6-9] and inflammatory pathways could be critical in the mechanisms underlying obesity and its complications, although the factors triggering the inflammatory response are not known, especially in humans [9]. The inflammatory process originates and resides mainly in adipose tissue, which produces cytokines as leptin and adiponectin [10,11]. This mechanism leads to reduction of adiponectin levels with increasing obesity, and to increase of C-reactive protein (CRP) and systemic inflammation [11,12]. Recent epidemiological studies have shown an association between obesity and functional bowel disorders [6,13,14], possibly as a consequence of low-grade inflammation [15]. An important and well described correlation exists between obesity and colo-rectal cancer (CRC) [6].

Intestinal inflammation is relatively rare in children compared to adults and the spectrum of aetiology is limited in childhood. We evaluated in a population of severely obese children the evidence and the degree of intestinal inflammation judged by non invasive tests. We also investigated the relationship between intestinal inflammation and glucose abnormalities.

## Patients and methods

Severely obese children (BMI >95% for age and sex, CDC), non compliant to the dietary regimen, were recruited at Department of Paediatrics, University of Naples Federico II, from January 1st 2004 to December 31st 2004.

The pubertal development stages were clinically assessed by the criteria of Marshall and Tanner [16] according to pubic hair and breast or genital development (Tanner's stage, TS). Body weight and height were measured in each child, and the BMI z-score (the number of SD units that the child's BMI deviates from the mean reference value for age and gender) relative to U.S. growth reference charts for 2000 [17], was calculated. The oral glucose tolerance test (OGTT) was performed with a load of 1.75 g/kg/body weight of glucose (maximum 75 gr) after a 12-hour overnight fast according to the World Health Organization recommendations. Plasma glucose and insulin levels were measured every 30 minutes for 120 minutes. The insulin value was considered normal if < 5.91 μU/ml [18].

Insulin sensitivity was estimated by the Homeostasis Model Assessment for Insulin Resistance (HOMA-IR) index from fasting glucose and insulin concentrations according to the following formula: insulin (mU/L) × glucose (mmol/L)/22.5 and referred to the normal values of HOMA-IR for children and adolescents assessed by d'Annunzio et al [19]. As the HOMA-IR index has been found very low in prepubertal children and increases with TS, we referred to the normal HOMA-IR values for TS [19].

Alterations of glucose metabolism were defined, according to the American Diabetes Association guidelines, as a fasting plasma glucose level between 100 and 126 mg per decilitre (impaired fasting glucose, IFG) and a two-hour plasma glucose level of 140 to 200 mg per decilitre (impaired glucose tolerance, IGT); type 2 diabetes was defined as a fasting glucose level of 126 mg/dl or higher or a 2-h plasma glucose level of 200 mg/dl or higher [20,21].

In all the patients enrolled, inflammatory bowel diseases (IBD) and systemic or intestinal infections were excluded during the evaluation.

CRP was the marker of systemic inflammation; it was considered abnormal if >0.5 mg/dl.

Rectal nitric oxide (NO) was measured in all patients by a dialysis bag, as described previously [22,23]. Briefly, a dialysis bag (3 cm long) was placed into the rectum and left for 30 min. The bag contained the following isotonic electrolyte solution: 20 mM KCl, 30 mM NaHCO3 and 110 mM NaCl. NO and its stable metabolites NO2^- ^and NO3^- ^were measured by the Griess reaction. Normal value was considered < 5 uM [23].

Faecal concentration of calprotectin was measured by a commercially available enzyme-linked immunosorbent assay (ELISA) test (Calprest Eurospital, SpA, Trieste, Italy). Normal concentrations of faecal calprotectin for adults range between 0 and 50 μg/g of wet stools, and values greater than 50 μg/g indicate intestinal inflammation [24]. These cut off levels are used for children older than 4 years [25] whereas the normal faecal concentration of calprotectin may be slightly higher in younger children [25]. However, faecal calprotectin values constantly higher than 100 μg/g are an alarm of intestinal inflammation.

## Statistical analysis

Statistical analysis was performed using SPSS software (SPSS, Chicago, IL, USA). Results are expressed as means ± SD or medians and range. Discrete values were expressed as percentage. A p value of less than 0.05 was considered significant. Linear correlation was used to evaluate the significance of the association between systemic and intestinal inflammation parameters and BMI z-score. ANOVA test was used to compare groups.

## Results

Thirty-four obese children (25 males and 9 females; median age 10.8 ± 3.4 yrs) were enrolled. Of the 25 boys, pubertal development was TS II in 22 and TS III in 3. Of the 9 girls, 3 were TS II, 2 were TS III and 4 were TS IV.

The BMI was >95° pc, ranging from 23 to 44 kg/m^2 ^and BMI z-score between 2.08 e 4.93, median 2.69 ± 0.53 (Table 1).

**Table 1:** Clinical features of 34 Severely Obese Children (25 males and 9 females).

	Overall (n = 34)	N G T (n = 10)	I G T (n = 24)	DM 2 (n = 7)
Age (yrs) (mean ± SD)	10.8 ± 3.4	9.6 ± 2.8	10.2 ± 3.9	9.7 ± 3

BMI (Kg/m^2^) (range)	23-44	25-40	23-44	23-42

BMI z-score (mean ± SD)	2.69 ± 0.53	2.5 ± 0.33	2.8 ± 0.65	2.56 ± 0.6

BMI: body mass index; NGT: Normal Glucose Tolerance; IGT: Impaired Glucose Tolerance; DM2: Diabetes Mellitus Type 2; SD: standard deviation.

**Figure 1:**
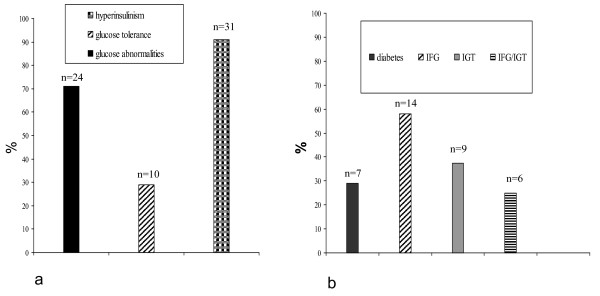
**Obese children with and without glucose abnormalities (n = 34)**. a: 71% with glucose abnormalities; 91% with hyperinsulinism b: Obese children with glucose abnormalities n = 24. 29% diabetes type 2; 58% impaired fasting glucose IFG; 37,5% impaired glucose tolerance IGT; 25% IFG/IGT.

**Figure 2:**
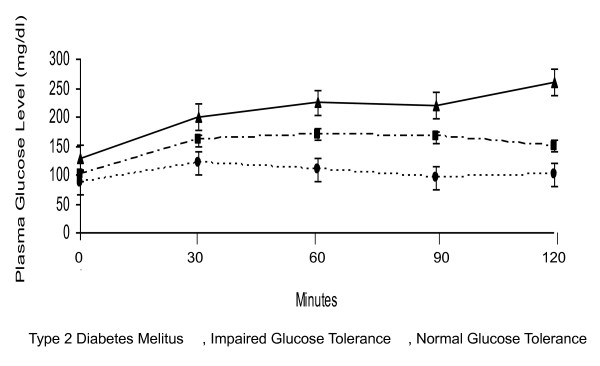
**Blood glucose levels during OGTT in obese children**. Mean (± SD) Plasma Glucose (mg/dl) during the Oral Glucose-Tolerance Test in obese children with Type 2 Diabetes Melitus, Impaired Glucose Tolerance or Normal Glucose Tolerance.

Glucose abnormalities were documented in 24/34 (71%) patients. In particular type 2 diabetes was observed in 7/24 patients (29%); impaired fasting glucose (IFG) in 14/24 patients (58%), impaired glucose tolerance (IGT) in 9/24 patients (37.5%). Six patients (25%) presented IFG and IGT. In 10/34 children (29%) there were no abnormalities of glucose metabolism (Figure 1, 2).

There was no difference in the prevalence of alterations of glucose metabolism in males and females and no alterations with respect to TS.

### Hyperinsulinism and Insulin resistance

Thirty-one patients (91%) were hyperinsulinemic (Figure 1). Mean insulinemia was 30.35 ± 38.7 μU/ml. It was pathological in 22/24 (92%) patients with glucose abnormalities, mean 36.36 ± 43.03 μU/ml, and in particular in 7/7 diabetic patients (100%).

A total of 30/34 patients (88.2%) had insulin resistance, defined by values compared with HOMA-IR index in healthy Italian children and adolescents, grouped by sex and TS [19]. The mean value of insulin resistance was 4.02 ± 5.8 for males TS II-III; 5.4 ± 8.2 for girls TS II-III; 7.9 ± 10.1 for girls TS IV. Values of HOMA-IR index were significantly higher in girls TS IV compared to males TS II-III and females TS II-III (p < 0.05). HOMA-IR index was abnormal in 22/24 (92%) patients with glucose abnormalities, mean 9.7 ± 12, and in all the diabetic patients.

### Systemic and intestinal inflammation parameters

#### CRP

**Figure 3:**
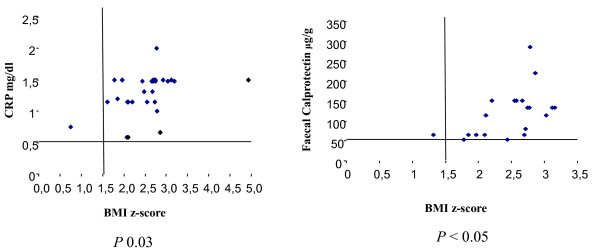
Linear correlation between BMI z-score and CRP and faecal calprotectin in 34 severely obese children.

CRP was increased in 25/34 (73.5%) obese patients, in particular in 5/7 (71%) with diabetes (mean 1.1 ± 0.53 mg/dl), indicating a low degree of systemic inflammation. Moreover, a statistical significant correlation was detected between BMI z-score and CRP >0.5 (p 0.03) (Figure 3).

### Faecal calprotectin

Faecal calprotectin was increased in 16/34 obese children (47%). It ranged from 15 to 270 μg/g with a mean value of 77 ± 68 μg/g, indicating a mild increase over normal. Individual values exceeded 100 μg/g in 12 patients (35%). Moreover, a significant correlation was detected between calprotectin and worsening obesity (BMI z-score) (Figure 3).

### Rectal Nitric Oxide

Median rectal nitric oxide was 6.8 ± 5 μM. It was pathological in 30/34 obese children (88%), in particular in 21/24 patients with alteration of glucose metabolism (87.5%, mean 6.5 ± 5), and in 7/7 diabetics (mean 8.9 ± 5.3).

## Discussion

The frequency of glucose abnormalities in our studied obese paediatric population is higher than other reported data. These abnormalities increase with worsening obesity.

Sinha et al described a prevalence of impaired glucose tolerance in 25% of obese children and 21% of obese adolescents and identified silent type 2 diabetes in 4% of this population [20]. Wiegand et al. found that 36.3% of Caucasian children and adolescents with obesity had impaired glucose tolerance and 5.9% type 2 diabetes [26]. In the present study we recruited very severely obese children and adolescents and the prevalence of hyperinsulinism, impaired glucose tolerance and diabetes type 2 appears largely higher than that described, being respectively 91%, 70.5% and 20.5%. In our opinion, this could be due to the higher risk characteristics of the studied population.

The vigorous hyperinsulinemic response to glucose found in the prediabetic stage in obese children and adolescents may reflect an up-regulation of beta-cell function caused by chronic severe insulin resistance [20]. Such a degree of hyperinsulinemia is not present in adults with impaired glucose tolerance [27,28]. The observation of hyperinsulinism in 92% of our patients with IGT confirm that insulin resistance is a strong predictor of the two-hour plasma glucose levels in youngsters, and the presence of high insulin levels in 100% of diabetics shows that beta-cell function was still preserved.

Alterations of glucose metabolism are related with markers of systemic and intestinal inflammation [29]. In this study an increased CRP (mean 1 ± 0.5) was present in more than 2/3 of patients with glucose abnormalities and in particular in 71% of diabetics (p < 0.05). We found a significant correlation between BMI z-score and CRP >0.5 (p 0.03), documenting a low grade systemic inflammation that increases with BMI in obese children.

Similarly, faecal calprotectin is elevated in obese adults and a correlation between CRP and calprotectin levels in this population is already described [6]. In our data, one third of children showed calprotectin concentrations above 100 μg/g and in 2 cases of very severe obesity the values were above 200 μg/g, almost reaching the levels measured in children with IBD [22]. We consider that these results, in the absence of specific intestinal symptoms and of intestinal diseases, and on the basis of faecal calprotectin and CRP levels intermediate between a normal intestinal mucosa and an active inflammation as an IBD, are an important evidence of chronic gut inflammation due to obesity also if on a small number of children. To our knowledge, the significance shown between BMI-z score and CRP and faecal calprotectin represents the first evidence of increased gut inflammatory activity in obese children otherwise normal, with an evidence of worsening intestinal inflammation with the grade of obesity.

Our data on rectal NO production support the calprotectin results. It has been reported that rectal NO production increases during active inflammation in children with IBD [22,23]. In this series, we found abnormal values of rectal NO in 88% of patients, in particular in 87.5% of those with glucose abnormalities and in 100% of diabetics. The increase in NO production in our children support the hypothesis that intestinal inflammation is a major feature in severe obesity and implicates the distal intestine in this process.

Findings in adult humans and in animals suggest that the inflammatory status at mucosal surfaces of various organs as adipose tissue, oesophagus, pancreas, colon [6,7], associated with the increase of fat mass, may be involved in the pathogenetic pathways of obesity complications.

In 2006 Sbarbati identified in a group of obese children, by ultramicroscopic analysis, the elementary "inflammatory" lesion of the adipose tissue consisting in a microgranuloma that evolves to fibrosis [30]. The lesion was not found in non-obese children and the extent of the lesion seemed to depend on the SD score of body mass index, proving that an "inflammatory" process exists in the adipose tissue of obese children and that it is an early alteration in humans [30]. Data regarding the histological lesion of the inflamed intestinal mucosa of obese children are lacking at the moment, but a similar pathway can be hypothesized. The evidence of inflammatory involvement of the distal intestine of obese children suggests an early onset of pathogenetic mechanisms that may lead to the complications of obesity as GI cancers. In this view, rectal NO and faecal calprotectin are useful non-invasive, reproducible and non-discomfortable tests to indirectly evaluate the intestinal status of severely obese children. Anyway, further research in this field is warranted.

## Abbreviations

BMI: Body Mass Index; CRC: Colo-rectal cancer; CRP: C-reactive protein; DM2: Diabetes Mellitus Type 2; GI cancers: Gastrointestinal cancers; HOMA-IR: Homeostasis Model Assessment for Insulin Resistance; IBD: Inflammatory Bowel Disease; IFG: Impaired Fasting Glucose; IGT: Impaired Glucose Tolerance; NGT: Normal Glucose Tolerance; NO: Rectal Nitric Oxide; TS: Tanner's stage.

## Competing interests

The authors declare that they have no competing interests.

## Authors' contributions

MIS conceived of the study and directed its design. MPC participated in the design of the study, performed the statistical analysis and drafted the manuscript. AF participated in the recruitment of the patients. MAC, VS and RM participated in the analysis of the data. LRA measured the rectal nitric oxide in all the patients. GV had a role in the design of the study. AG coordinated the study and the people involved in it. All authors read and approved the final manuscript.
